# Intensivists’ perceptions of what is missing in their compassionate care during interactions in the intensive care unit

**DOI:** 10.1186/s12913-022-08584-0

**Published:** 2022-09-22

**Authors:** Shahla Siddiqui, Enas Mohamed, Balachundhar Subramaniam, Hibiki Orui, Michael Nurok, Miguel Angel Cobas, Mark E. Nunnally, Christiane Hartog, Raanan Gillon, Beth A. Lown

**Affiliations:** 1 Department of Anesthesia, Critical Care and Pain Medicine, Beth Israel Deaconess Medical Center, Harvard Medical School, 330 Longwood Ave, MA Boston, USA; 2 Department of Anesthesia, Critical Care and Pain Medicine, Beth Israel Deaconess Medical Center, Harvard Medical School, Boston, USA; 3 Sadhguru Center for a Conscious Planet- Enhancing Consciousness, Cognition, Compassion, Beth Israel Deaconess Medical Center, Boston, MA USA; 4 Cardiac Surgery Intensive Care Unit, Department of Cardiac Surgery, Fellowship in Critical Care Medicine, Department of Anesthesiology, Smidt Heart Institute | Cedars-Sinai Medical Center, Los angeles, CA USA; 5 Department of Anesthesiology, Society of Critical Care Anesthesiologists, Anesthesiology Steering section Society of critical care medicine, University of Miami Miller School of Medicine, Miami, FL USA; 6 Department Of Anesthesiology, Perioperative Care and Pain Medicine, Neurology, Surgery and Medicine, Adult Critical Care Services, New York University Lagone, New York, NY USA; 7 Department of Anesthesiology and Operative Intensive Care Medicine, Charité Universitaetsmedizin Berlin, Berlin and Klinik Bavaria, Kreischa, Germany; 8 Imperial College, London, UK; 9 The Schwartz Center for Compassionate Healthcare, Harvard Medical School, Boston, MA USA

**Keywords:** Compassion, Compassionate care, Communication, Education

## Abstract

**Background:**

We proposed that the behaviors that demonstrate compassionate care in the intensive care unit (ICU) can be self-assessed and improved among ICU clinicians. Literature showing views of intensivists about their own compassionate care attitudes is missing.

**Methods:**

This was an observational, prospective, cross-sectional study. We surveyed clinicians who are members of professional societies of intensive care using the modified Schwartz Center Compassionate Care Scale® (SCCCS) about their self-reported compassionate care. A modified SCCCS instrument was disseminated via an email sent to the members of the Society of Critical Care Medicine and the European Society of Intensive Care Medicine between March and June 2021.

**Results:**

Three hundred twenty-three clinicians completed the survey from a cohort of 1000 members who responded (32.3% response rate). The majority (54%) of respondents were male physicians of 49 (+ − 10 SD) years of age and 19 (12 + − SD) years in practice. The mean SCCCS was 88.5 (out of 100) with an average score of 8 for each question (out of 10), showing a high self-assessed physician rating of their compassionate care in the ICU. There was a positive association with age and years in practice with a higher score, especially for women ages 30–50 years (*P* = 0.03). Years in practice was also independently associated with greater compassion scores (*p* < 0.001). Lower scores were given to behaviors that reflect understanding perspectives of families and patients and showing caring and sensitivity. In contrast, the top scores were given to behaviors that included conducting family discussions and showing respect.

**Conclusion:**

Physicians in the ICU self-score high in compassionate care, especially if they are more experienced, female, and older. Self-identified areas that need improvement are the humanistic qualities requiring sensitivity, such as cognitive empathy, which involves perspective-taking, reflective listening, asking open-ended questions, and understanding the patient’s context and worldview. These can be addressed in further clinical and ICU quality improvement initiatives.

## Introduction

‘Compassion’ is defined as “the recognition and acknowledgement of others’ distress and suffering coupled with motivation that drives action to alleviate it.” [1] Compassionate care is central to all healthcare, not just to end of life care in the intensive care unit (ICU). Compassionate care is highly valued by both patients and clinicians [2] and enables equitable access to health care that is relationship-centered, safe, and effective as a human right. All patients and staff can benefit from relationship-centered approaches that enhance the understanding of medical treatment with clear and sensitive communication [3]. For those who suffer and those who witness this suffering in the ICU, compassion is fundamental to the purpose of healthcare and medicine [4]. The importance of compassionate care in the ICU has been highlighted during the current Coronavirus disease 2019 (COVID-19) pandemic. Its importance has come under the spotlight in mitigating the stress of working in the ICU, showing empathy towards critically ill patients as well as showing compassion to families who have not been able to be at the bedside of their loved ones due to the pandemic restrictions [5].

It has been difficult to define, improve and teach compassionate care behaviors by clinicians in the ICU [6, 7]. Researchers have identified a discord on how compassionate care is perceived by clinicians, and how it is recognized and appreciated by patients and families who are the stake holders in the ICU environment [8, 9]. Challenges to compassionate care in the ICU include a focus on technology and data rather than the human element of care, such as gaps in interprofessional communication, workplace-related conflict, resource constraints, personal life stressors and discord between patients, families and clinicians [10]. Patients and families have reportedly felt that they have not been given compassionate care when interacting with healthcare personnel [11]. There is also the important consideration of feeling overwhelmed emotionally when displaying compassion and sensitivity with acutely ill patients and their family members [12]. There exists a dearth of literature on how compassionate care is perceived by clinicians working in the ICU and caring for patients who are critically ill and their families [13].

We hypothesize that compassionate care in the ICU is demonstrated by a set of behaviors that can be self-assessed and potentially improved by ICU physicians. Our aim was to understand intensive care clinicians’ (ICU physicians’ and nurses’) perceptions of their compassionate care and to identify behaviors that could be modelled, learned and improved.

## Methods

### Measurements

Institutional review board approval was obtained from the Beth Israel Deaconess Medical Center IRB (2021P000065). We used the Schwartz Center Compassionate Care Scale® (SCCCS) [14] to assess the compassionate care provided by professional caregivers [15]. A version of this instrument has been used by researchers to assess physician compassion as assessed by recently hospitalized [16] and ambulatory patients [14], as well as by physicians (non-intensivists) [2] and nurses [17] to assess self-reported compassionate care in previous studies. This was an observational, prospective, cross-sectional study, using the SCCCS. The psychometric characteristics of the SCCCS have been evaluated as a clinician and patient self-reported assessment of compassionate care and have illustrated excellent internal consistency and test-retest validity [14]. It is a unidimensional scale that correlates with patients’ general and emotional satisfaction with their care experiences [16]. We modified the 12-item SCCCS scale by removing 2 questions related to time-sensitivity and speaking directly with the patient (‘Communicates test results in a timely and sensitive manner’ and ‘spends enough time with you’) and adding a question about conducting end-of-life discussions (Conduct end-of-life communication with empathy and patience). The scale asks clinicians to rate their behaviors on a scale of 1–10, with 1 indicating ‘not at all successfully’ and 10 indicating ‘very successfully.’The modified tool was reviewed by the intensivist authors for content validity and relevance. This survey was distributed via Research Electronic Data Capture (RedCap) a secure web application for building and managing online surveys and databases [18]. Participants were sent a link to the survey via email and invited to participate in this anonymous 10-minute study. The study period was March to June 2021.Three email reminders were sent over the survey period with the link as stipulated in the IRB. Respondents were asked about their age, gender, profession, region of and years in practice. The participants were then asked to respond to the 11-item modified SCCC scale.

### Study population

This survey was distributed to the membership of the United States Society of Critical Care Medicine (SCCM) and the European Society of Intensive Care Medicine (ESICM). The SCCM and ESICM membership spans across the world and is open to all professions within the ICU, such as physicians, nurses, pharmacists, respiratory therapists, and others. Emails were sent to those members who had indicated in their profile that they were willing to receive such survey requests. The survey was disseminated by the administrative staff of the respective societies and the survey included an initial informed consent prompt following which the participant could proceed with the survey.

### Statistical methods

Descriptive analysis was used for the demographics. Categorical variables are presented as valid counts and percentages. Linear regression was used to evaluate the association between the average SCCC scale, gender, age, and years in practice. Six age groups were studied for 10 year intervals each. *P* values less than 0.05 were considered statistically significant. The Spearman rank correlation coefficient was calculated for measuring the association between years in practice and the average SCCC scores. Statistical analyses were performed using SAS software, version 9.1 of the SAS System for windows. Copyright© 2016 SAS Institute Inc.

## Results

### Demographics

Three hundred twenty-three participants consented to participate and completed the survey (32.3%). Table 1 shows the demographics of the participants; 53.8% (174 respondents) were male, 96.9% (313 respondents) were physicians, with a mean age 49 years (+ − 10.3 SD) and 19 years (+ − 12.3 SD) in practice. All responses were included in the analysis. Although the remaining non-physician participants were nurse members of the critical care societies, they were involved in ICU decision making and clinical care.

**Table 1:** Demographics

Demographic variable	Total Participants, *n* = 323
Gender, No. (%)	
Male	174 (53.87)
Age, Mean (SD)	49.24 (10.35)
Profession, No. (%)	
Physician	313 (96.9)
Nurses	10 (3.1)
Years in Practice, Mean (SD)	19.32 (12.3)
Region of participants, No. (%)	USA 206 (64%)
	Europe 103 (32%)
	Asia 14 (4%)

### Overall compassion scores and individual questions

Table 2 shows the SCCCS responses on a scale of 1 to 10. The table shows the average score for each question, percentage of responses with a score of 9 or above, and percentage of responses with a score of 10 (“top box scores”), consistent with methods used for public reporting of U.S. Hospital Consumer Assessment of Healthcare Providers and Systems [19] (HCAHPS) scores. Top Box scores are the best or highest scores rated in a survey [20]. Total average score was 88.5/100 and the average individual response was 8/10, showing a high self-reported score. The average scores varied between 7 and 9. The lowest average score by the participants was 7 and involved ‘understanding the effect of the patient’s illness on them and their families’. Average scores were higher (8, 8.5 and 9) for questions that included conveying information, acting with respect, and conducting end-of-life discussions with empathy and patience.

**Table 2:** Compassion Scale Responses: ‘top box score’(*) questions

Questions	M (SD)	Score of 9 or above, No. (%)	Score of 10, No. (%)
Express sensitivity, caring and compassion for your ICU patients	7.5 (2.12)	129 (40.2)	16 (19)
Strive to understand your patient’s or their family’s emotional needs	7.5 (2.12)	151 (46.9)	60 (18.6)
Consider the effect of your ICU patient’s illnesses on them and their family	7.0 (2.83)	143 (44.9)	65 (20.4)
*Listen attentively to your patient or their loved ones	8.0 (2.82)	146 (45.5)	59 (18.4)
*Convey information in a way that is understandable	8.5 (2.12)	184 (57.9)	79 (24.8)
*Gain the trust of your patient and their family	8.5 (2.12)	152 (47.4)	66 (20.6)
Involve patients and their families in decisions about the patient’s treatment	7.5 (0.7)	140 (43.7)	66 (20.6)
*Comfortably discuss sensitive, emotional or psychological issues	8.5 (2.12)	146 (45.7)	77 (24.1)
*Treat the patient as a person not just as a disease	8 (1.41)	200 (62.5)	98 (30.6)
*Show respect for your patient and their family	8.5 (0.70)	222 (69.1)	99 (30.8)
*Conduct end-of-life communication with empathy and patience	9 (1.41)	229 (71.6)	113 (35.3)
Average Total Schwartz Score (Out of 110)	88.5		
Average Individual Responses (On a scale of 0 to 10)	8.045		

### ‘Top box’ scores (higher scores of 9 or 10)

Analysis of the “top box” scores showed that less than 50% of respondents rated themselves 9–10/10 on behaviors that require listening, eliciting, and processing information about patients’ concerns and emotional needs. Only 18.4% gave themselves the highest score 10/10 on ‘listening attentively’. However, more than 50% of respondents rated themselves 9–10/10 on expressive acts of compassion (e.g., showing respect, treating the patient as a person). The highest “top-box” rated skill was empathically conducting end-of-life discussions.

### Regression analysis

**Fig. 1:**
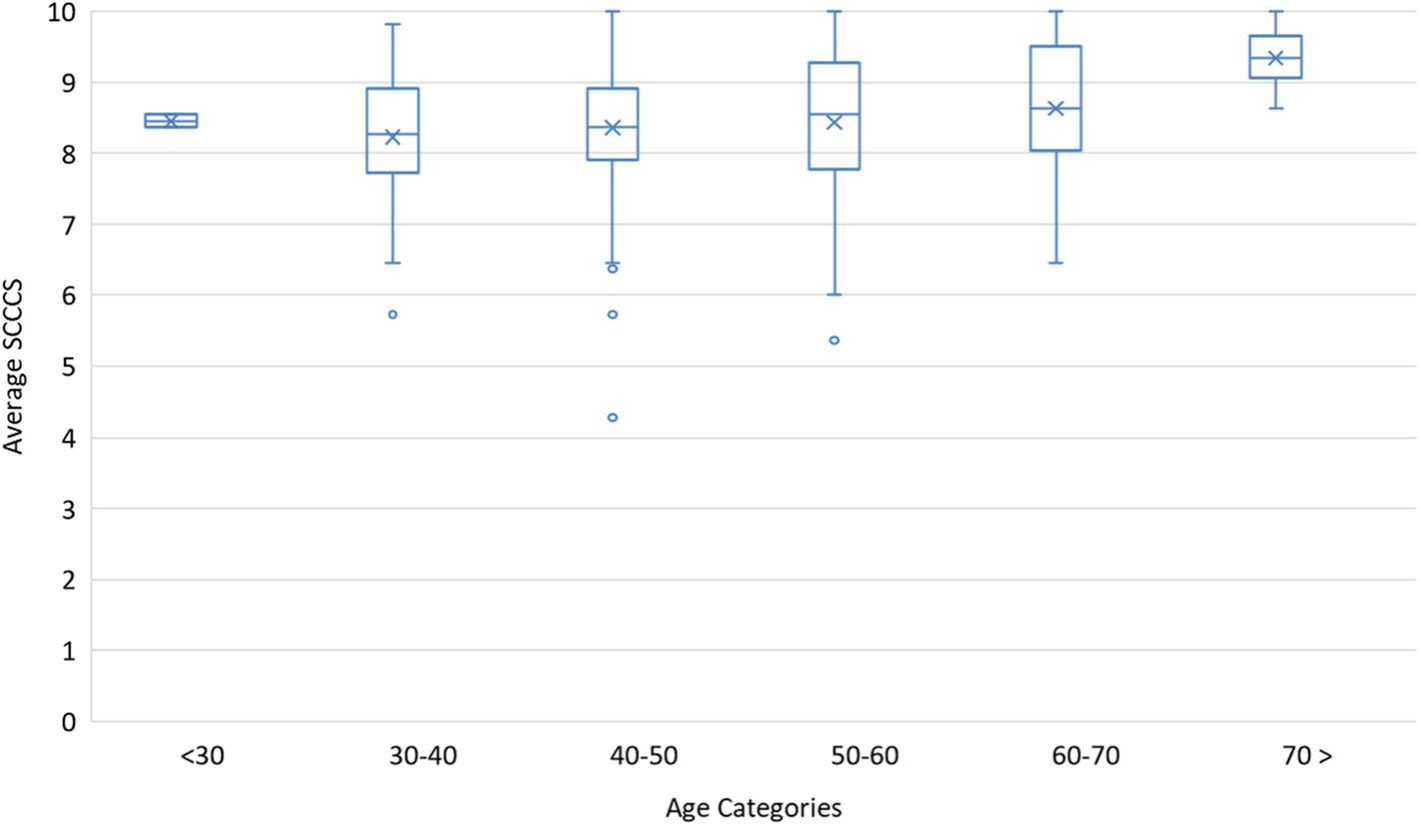
Average SCCCS with increasing age categories (in years)

**Fig. 2:**
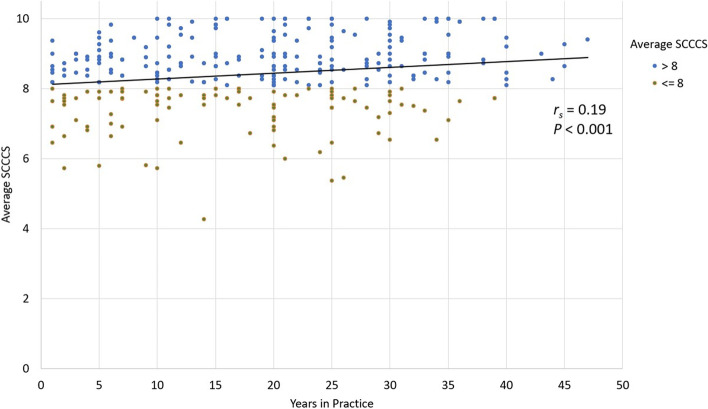
Association between average SCCCS and years in practice (r_s_ = Spearman rank correlation)

Table 3 shows the inferential associations using multiple linear regression adjusted for age and gender. There was suggestive evidence that the average compassion score trends higher with increasing age, but this did not reach statistical significance (*P* = 0.07). We did, however, see significantly higher average compassion scores with increasing age when controlling for gender in the adjusted model, especially for the 3rd decade (*P* = 0.03) **(**Fig. 1**).** Females between 30 and 50 years old reported higher average compassion scores than in males**.** There was a significant association between a higher self-reported compassion score and years in medical practice (*P* = 0.001). From the linear regression, we observe 0.017 point increase in the average SCCCS for each additional year in practice (*P* < 0.001) **(**Fig. 2**).** The Spearman rank correlation coefficient was calculated for this relationship and found to be r_s_ = 0.19 (*P* < 0.001).

**Table 3:** Analysis of SCCCS and age and gender in decades

	N	Adjusted Model^a^		
		Estimate	***P***-Value	Type III ***p***-value
Age category				0.033
< 30	5	−1.13	0.052	
30–40	64	−1.19	0.003	
40–50	99	−0.96	0.015	
50–60	95	−0.93	0.018	
60–70	51	−0.72	0.073	
> 70	7	Ref.	–	
Gender				
Female	147	0.19	0.107	0.107
Male	174	Ref.	–	

^a^Model adjusted for gender

## Discussion

ICU clinicians rate their compassionate behaviors highly and this is correlated with increasing age, female gender and years of experience. They also rate themselves better at demonstrating compassion actively, such as holding meetings, conducting end-of-life meetings with empathy, showing respect, and conveying information. This study also identified barriers to compassion in healthcare, the most significant being able to understand and display emotional sensitivity. The scores were lower for behaviors that involve elicitation, listening, processing and handling of patients’ emotions, such as considering the effect of the illness on their patients and their families, understanding patients’ and families’ emotional needs, involving families in decisions and comfortably discussing sensitive emotional or psychological issues with them.

Skills that present an opportunity to strengthen care behaviors include elicitation and expressive skills. Cognitive empathy involves active listening, asking open ended questions, contextualization, and taking a larger perspective [21]. These are elicitation skills that will enrich cognitive empathy. A recent study by Vasher, et al. suggested that the self-assessment of clinicians who lack communication skills did not agree with those of external-intensivist raters and they may also lack the “metacognitive skills required to recognize their deficiencies.” [22] Expressive skills such as acknowledgment, partnership, and validation of emotions convey empathic concern and compassion [23]. While respondents rated their skills highly on average, their top-box scores are contrary to this finding, as most participants were able to grade the nuances of their compassionate care and highlight areas where further enhancement was needed. These skills can be targeted to improve the expression of compassion in ICU physicians. Compassion aptitude is strongly influenced by the inherent qualities that healthcare workers possess at baseline, however, it is important to train physicians in the emotional aspects of eliciting and responding to emotions in expressing compassion in the ICU where compassionate care is essential [24].

To harness emotions and provide comfort may not come intuitively to many, and neither is it generally explicitly taught. Self-efficacy in the provision of compassionate care motivates one to offer and potentially improve it [25]. Self-efficacy is widely perceived as an important motivator of behavior change. Perhaps if ICU clinicians are shown to be able to model and improve their own ‘compassion skills’ this will motivate others to improve their compassionate behaviors towards patients and families in the ICU [26]. Halpern and colleagues recognize that emotional resonance is important in teaching clinical empathy, rather than simply teaching cognitive comprehension [27]. ‘Emotional regulation’ is also an important skill which can be self-taught and role modelled in the ICU. Overidentifying with the patient can potentially lead to burnout from being empathically overwhelmed [28]. This can be counterbalanced by learning how to regulate ones’ emotions and actually support the patient rather than emotionally withdrawing due to feelings of empathic distress. Another approach to ‘compassion training’ has been described by Klimecki and Singer [29]. There has been an increase in the rate of burnout among the more ‘caring’ professions such as critical care [12, 30]. However, by means of this training, emotional self-regulation and human connectivity has been proven to mitigate burnout and increase job satisfaction [31]. This capacity to self-regulate can be learned through cognitive behavioral empathy and mindfulness training and both are useful tools for clinicians to acquire [32].

Limitations of the study include the relatively limited response rate which is inherent in survey methodology. However, a 30% response rate is a generally acceptable rate. Modification of the validated tool for the ICU environment was done with permission of the Schwartz Center for Compassionate Healthcare and was tested for content validity within the authorship. Another limitation may be that the study may have only captured respondents who are more self-reflective in their practice or more likely to rate their care as compassionate and not be representative of the general intensivist population. Our study was also skewed towards physician members of the societies, but this may be the general proportion of membership as well.

This study highlights behaviors that could be targets for learning and improvement; i.e., empathic understanding through attentive listening and perspective-taking, and the behavioral expression of empathic concern and caring. The educational and quality improvement challenge lies in translating these behaviors into concrete educational initiatives as required competencies [33]. Patient and family feedback after encounters could be an important tool to complete the loop for self-learning. A comparison of patients’ or family surrogates’ compassion ratings with self-reported ratings of compassion may be helpful in pointing out gaps in self-assessment and areas for improvement. A further possible improvement might be to ask participants to provide a range of ratings of the different compassion (or lack of compassion) behaviours that they have personally encountered in their practice.

The absence of studies of compassionate interactions within ICU populations suggests that this area warrants further research. Our study presents opportunities for a change in physician self-assessment, self-improvement and self-management of their compassionate care skills, and therefore for potential reduction in the risk of burnout in the ICU.

## Conclusion

Intensivists rate their compassion as high in most domains assessed, however, we have identified a few specific domains that intensivists self-rate lower than others. These may be areas to develop further with education or quality improvement efforts. Reflective listening skills and empathic understanding are two areas identified as needing development that can be taught and improved. The incorporation of these skills can help form relationships with patients and their families, build trust, and allow compassionate behaviors and communication in difficult situations such as breaking bad news and conflict resolution, without the risk of compassion fatigue or detachment. Future research must also incorporate the perspective of patients and their families, whose perception is integral in understanding their expectations of the healthcare experience.

## Data Availability

The datasets generated and/or analysed during the current study are not publicly available due to IRB regulations, but are available from the corresponding author on reasonable request.
